# Phytochemical Profile of Brown Rice and Its Nutrigenomic Implications

**DOI:** 10.3390/antiox7060071

**Published:** 2018-05-23

**Authors:** Keneswary Ravichanthiran, Zheng Feei Ma, Hongxia Zhang, Yang Cao, Chee Woon Wang, Shahzad Muhammad, Elom K. Aglago, Yihe Zhang, Yifan Jin, Binyu Pan

**Affiliations:** 1 Faculty of Food Science and Nutrition, Universiti Malaysia Sabah, Kota Kinabalu 2073, Sabah, Malaysia; rkeneswary@yahoo.com; 2 Department of Public Health, Xi’an Jiaotong-Liverpool University, Suzhou 215123, China; Yifan.Jin14@student.xjtlu.edu.cn; 3 School of Medical Sciences, Universiti Sains Malaysia, Kota Bharu 15200, Kelantan, Malaysia; 4 Department of Food Science, University of Otago, Dunedin 9016, New Zealand; zhanghongxia326@hotmail.com; 5 Department of Health Promotion, Pudong Maternal and Child Health Care Institution, Shanghai 201399, China; evacaoyang@163.com; 6 Department of Biochemistry, Faculty of Medicine, MAHSA University, Bandar Saujana Putra 42610, Jenjarom, Selangor, Malaysia; wang.chee@mahsa.edu.my; 7 Institute of Basic Medical Sciences, Khyber Medical University, Peshawar 25100, Pakistan; shahzad.ibms@kmu.edu.pk; 8 Joint Unit of Research in Nutrition and Food Science, Ibn Tofail University, Kenitra 14000, Morocco; aglagoelom@gmail.com; 9 Division of Medicine, School of Life and Medical Sciences, University College London, London WC1E6BT, UK; yihe.zhang.16@ucl.ac.uk; 10 Department of Clinical Nutrition, The First People’s Hospital of Wujiang District, Suzhou 215200, China; panbinyu19881102@126.com

**Keywords:** brown rice, nutrigenomics, phenolics, rice

## Abstract

Whole grain foods have been promoted to be included as one of the important components of a healthy diet because of the relationship between the regular consumption of whole-grain foods and reduced risk of chronic diseases. Rice is a staple food, which has been widely consumed for centuries by many Asian countries. Studies have suggested that brown rice is associated with a wide spectrum of nutrigenomic implications such as anti-diabetic, anti-cholesterol, cardioprotective and antioxidant. This is because of the presence of various phytochemicals that are mainly located in bran layers of brown rice. Therefore, this paper is a review of publications that focuses on the bioactive compounds and nutrigenomic implications of brown rice. Although current evidence supports the fact that the consumption of brown rice is beneficial for health, these studies are heterogeneous in terms of their brown rice samples used and population groups, which cause the evaluation to be difficult. Future clinical studies should focus on the screening of individual bioactive compounds in brown rice with reference to their nutrigenomic implications.

## 1. Introduction

For centuries, rice (*Oryza sativa* L.), one of the most well-known cereal foods, has been a primary food for many people around the world and is known to feed half of the population [1]. Therefore, the role of rice as a staple food in providing nutrition to populations has been acknowledged. In 2015, the global rice paddy production was 739.1 million tonnes, yielding 490.5 million tonnes of white rice after milling. The rice paddy production in Asia was 668.4 million tonnes, accounting for 90% of the global production, indicating that rice consumption occurs mostly in Asian countries. The environmental flexibility of culturing rice paddies at various temperatures, humidities and soil conditions allows rice to become a globally-viable crop [2]. However, the health benefits of rice were never considered because rice is considered as a staple food based on the palatability and availability. The major producers of rice are China, India and Indonesia [3].

There are more than 8000 varieties of rice, which have different types of quality and nutritional content. After the post-harvest process, all the varieties of rice can be categorised as either white or brown rice [4]. The aromatic rice varieties, known collectively as “Basmati rice”, have been sourced by people from Asian and European countries, because aroma has been considered as the highest preferred characteristic of cereal grain. Basmati rice possesses unique cereal quality features, such as long, supreme grains, characteristic aroma, swelling on cooking and tenderness of cooked rice. Basmati rice with a high amylose to amylopectin ratio and a medium glycaemic index is suitable for staple diets of diabetics [5].

Rough rice can be separated into husk and brown rice through a threshing process. The components in brown rice that was hulled from rough rice are bran layers (6–7%), an embryo (2–3%) and an endosperm (about 90%) [6]. Brown rice can be further separated into polished rice, commonly called white rice, which is obtained by removing the bran. Minor differences may exist in the degree of milling. Brown rice has a nutty flavour, chewier than white rice, but more easily goes rancid, as well [7]. The difference between brown rice and white rice can be obtained through milling [7]. White rice contains mainly the starchy endosperm. The removal of rice bran leads to a loss of nutrients. During milling, about 85% of the fat, 15% of protein, 75% of phosphorus, 90% of calcium and 70% of B vitamins (including B_1_, B_2_ and B_3_) are removed [7].

As the degree of milling increases, the loss of phytochemical compounds beneficial to health occurs, and cellular antioxidant activity decreases. Furthermore, the contents of phenolic compounds have also been shown to decrease by increasing the degree of milling. Thus, by carefully controlling the degree of milling during rice processing, both the sensory quality and nutritional composition could be optimized. Thus, brown rice with a low degree of milling (<2.7%) exhibits a more ideal balance between sensory quality and retention of beneficial phytochemicals [8]. Brown rice is a rich source of various bioactive compounds, such as γ-oryzanol, tocopherol, tocotrienol, amino acids, dietary fibres and minerals. It is less consumed than white rice because its cooking is more difficult than white rice due to its slow water absorption, and the palatability quality of brown rice is inferior to white rice [9].

There are two types of brown rice, which are germinated and non-germinated. Germinated brown rice is obtained by immersing the brown rice grain in water to initiate germination [10]. The benefits of germinated brown rice are that the nutrients found in brown rice are more easily digested and the texture of brown rice is better [10]. Germination has been employed to improve the texture of cooked brown rice. It also initiates numerous changes in the composition and chemical structure of the bioactive components. Germination could induce the formation of new bioactive compounds, such as gamma-aminobutyric acid (GABA). The consumption of germinated brown rice is increasing in many Asian countries because of its improved palatability quality and potential health-promoting functions [11].

Advances in the human genome era have shown that diet plays an important factor in the health and the causation chronic diseases such as type 2 diabetes. This is because the diet-genome interactions can result in changes especially in the proteome, transcriptome and metabolome. For example, current healthcare practitioners recommend brown rice to be consumed rather than white rice. This is due to the fact that brown rice is more nutritious. One common trait between white rice and brown rice is that they are both gluten free and contain no trans fat or cholesterol [7]. Encouraging people to eat brown rice more is a difficult challenge due to its taste, which is less likeable compared to the taste of white rice [7]. In the United States, more than 70% of rice consumed is white rice, and rice consumption has reached 9.3 kg per capita since the 1930s [1]. In addition, the consumption of brown rice is beneficial for postprandial blood glucose control because brown rice has a lower glycaemic index than white rice (55 vs. 64) [12].

Rice is the main staple food for more than half of the world’s population. The cereal was also utilised as a popular remedy since ancient times for several therapeutic purposes. Rice or rice-based products were also well documented in the traditional medicines of different Asian countries. The well-known popular uses are anti-diabetic, anti-inflammatory for the airway, ailment of gastrointestinal disorders and diarrhoea, diuretic, source of vitamins and skin preparations [13,14]. One of the rice varieties, red rice Rakthashali, is a staple food in India and has been described by Ayurveda practitioners as a functional food for a number of medications [15]. The medicinal rice Kullakar has high thiamine content, while the Karikalaveya variety is high in riboflavin and niacin [16].

Therefore, the aim of our work is to review the phytochemical constituents and nutrigenomic implications of brown rice in relation to animal and human studies. In addition, our work has also contributed significantly to the current understandings of brown rice with reference to the nutrigenomic implications of brown rice shown in human intervention studies. Therefore, this mini-review will provide a valuable reference resource for future studies in such areas.

### Search Strategy

An electronic literature search was conducted using PubMed, Medline (OvidSP) Cochrane CENTRAL and Web of Science until December 2017. Additional articles were identified from references in the retrieved articles. Search terms included combinations of the following: rice, brown rice, phytochemicals, nutrigenomics and bioactives. The search was restricted to articles in English that addressed the phytochemical constituents and nutrigenomic implications of brown rice.

## 2. Phytochemical Compounds in Brown Rice

**Figure 1:**
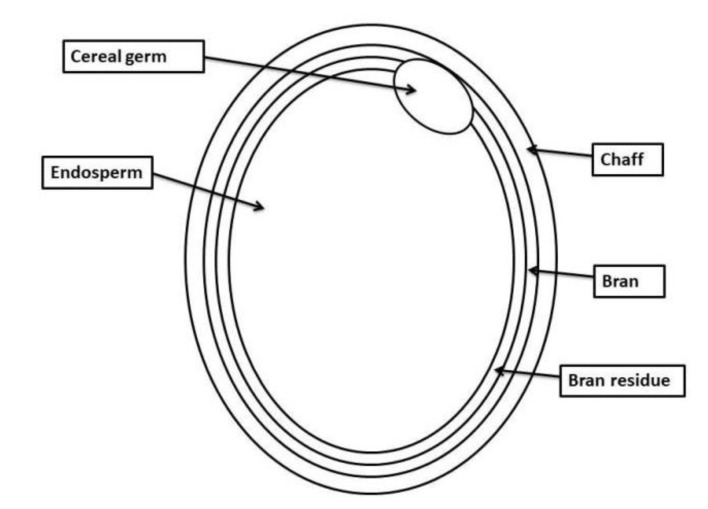
The various parts of the rice grain.

The advantages for health with the consumption of brown rice mainly come from the phytochemicals found in its bran layers [17]. Figure 1 shows the various parts of the rice grain. The phytochemical composition of brown rice cannot be dissociated from the scientific work of the Dutch Nobel prize scientist Christiaan Eijkman who initially reported the potential of brown rice and the story behind beriberi in humans in the previous centuries. Table 1 show the major phytochemical composition of brown rice. In addition to B vitamins, phytochemicals found in brown rice include dietary fibre, functional lipids, essential amino-acids, phytosterols, phenolic acids, flavonoids, anthocyanins, proanthocyanins, tocopherols, tocotrienols, minerals, gamma aminobutyric acid (GABA) and γ-oryzanol [11,17]. Brown rice also contains high levels of phytic acid [18].

Amongst these nutritional factors, phenolic acids are the most common substances found in brown rice [19]. Phenolics are classified under phytochemicals having one or more aromatic rings with one or more hydroxyl groups [20]. Phenolic compounds are associated with diverse human health benefits including anti-inflammatory, hypoglycaemic, anticarcinogenic, antiallergenic and antiatherosclerotic properties [20]. Examples of phenolics are phenolic acids, flavonoids, tannins, coumarins and stilbenes [21]. In rice, phenolics are found in three distinct forms, which are free, soluble-conjugated and bound forms, and the bound form is the main form among the three [22]. High levels of phenolics exist in the germ and bran layers [23]. Since brown rice does not undergo any polishing or milling, these phenolics found in the germ and bran layers are easily preserved. Free phenolics are the most readily available for absorption in the small intestine, while bound phenolics tend to be preserved throughout the intestinal tract and released in the bowel, where they interact with the microbiome to favour the Firmicutes/Bacteroidetes ratio [24]. The two main groups of phenolic acids are p-hydroxybenzoic acid and p-hydroxycinnamic acid derivatives [21]. The primary phenolic compound found in brown rice is *trans*-ferulic acid (range: 161.42–374.81 μg/g), a hydroxycinnamic acid that exists in the bound form [19]. The second major phenolic acid found in brown rice is *trans*-*p*-coumaric acid (range: 35.49–81.52 μg/g), which is a hydroxycinnamic acid derivative with its bound form making up about 98% [19]. Another component that is widely found is *cis*-ferulic acid (range: 20.76–83.02 μg/g), an isomer of *trans*-ferulic acid, which is found abundantly in the bound form [19].

Soluble phenolic compounds consist of free phenolic acids and hydroxycinnamate sucrose esters [25]. The main soluble phenolic compounds in brown rice are feruloylsucrose, sinapoyl sucrose and ferulic acid [25]. The components that are notable in brown rice in bound forms are 8-*O*-4′ diferulic acid (DFA) (range: 13.88–22.61 μg/g), 8-5′ benzofuran DFA (range: 9.28–14.79 μg/g), 5-5′ DFA (range: 7.29–13.86 μg/g) and 8-5′ DFA (range: 3.26–8.79 μg/g) [19]. The three other hydroxycinnamic acid derivatives that were found in brown rice in small amounts are caffeic acid (range: 0.00–1.44 μg/g), sinapic acid (range: 1.19–1.25 μg/g) and chlorogenic acid (0.63 μg/g) [19]. Two examples of hydroxybenzoic acid derivatives that were found are vanillic acid (range: 2.65–4.74 μg/g) and syringic acid (range: 0.47–2.52 μg/g) [19]. In brown rice, catechin (range: 4.06–8.92 μg/g), quercetin (range: 3.27–6.53 μg/g) and kaempferol (range: 1.30–3.04 μg/g) are the three main flavonoids that are usually found in the free form [19].

In contrast to white rice, brown rice is still constituted by the germ and the bran layers, which contain diverse nutritional compounds, including anti-oxidants [26]. Therefore, despite its high nutritional value, brown rice is consumed less than white rice mainly due to its appearance, longer cooking time, cost, limited availability and bioavailability and poor appreciation of its nutritional value [27]. Apart from cooking, several approaches, including germination, have been emphasized to improve the palatability and the bioavailability of the nutrients present in brown rice. Germination improves the texture and the bioavailability of the nutrients and the phytochemicals [25,28].

In germinated (sprouted) brown rice, about a 70% drop is found in feruloylsucrose (from 1.09–0.27 mg/100 g of flour) and sinapoyl sucrose (from 0.41–0.13 mg/100 g of flour), whereas free ferulic acid content increased (0.48 mg/100 g of flour) when compared to brown rice [25]. However, in general germinated brown rice contains less soluble phenolic compounds when compared to brown rice (1.45 vs. 2.17 mg/100 g of flour) [25]. Apart from that, the sinapinic acid level also increases ten-fold in germinated brown rice (0.21 mg/100 g of flour) compared to brown rice (0.02 mg/100 g of flour) [25]. Germination also increases the levels of the GABA in brown rice [28]. Inositol hexaphosphate is a naturally-occurring molecule found in brown rice [18]. This compound has demonstrated anti-cancer properties [18]. Selenium is a trace mineral, which is found abundantly in brown rice [29]. The function of selenium is to induce DNA repair and combine in damaged cells to promote apoptosis, which is the self-destruction of the cells in the body to remove damaged and worn out cells [29]. Selenium also functions as a cofactor of glutathione peroxidase, which is an enzyme used in the liver to detoxify many possible harmful molecules [29]. Plant lignans are one type of phytonutrient that is found widely in brown rice, which are then converted to mammalian lignan, called enterolactone [30]. Brown rice also serves as a rich source of magnesium. Magnesium plays an important role in our body, as it works as a cofactor of more than 300 enzymes [31]. About 21% of the daily value of magnesium can be obtained by consuming a cup of brown rice [31].

Brown rice contains a high amount of dietary fibre, which has been shown to protect against colorectal cancer [32] and breast cancer [33]. In an animal study, rice bran from brown rice was shown to be beneficial against the development of polyps in the bowel [34]. Due to its high content in fibre, brown rice has a lower glycaemic index, compared to white rice [12]. Consumption of brown rice compared to white rice results in improved endothelial function, without changes in HbA1c levels, possibly through reducing glucose excursions [35]. Vitamin E is also found in brown rice, mainly in two types of structure, which are tocopherols (α, β, γ and δ forms) and tocotrienols (α, β, γ and δ forms) [17]. The function of vitamin E is antioxidant activity, maintenance of membrane integrity, DNA repair, immune support and metabolic processes [21].

Regarding insoluble phenolic compounds, germinated brown rice has at least twice the total content value of insoluble phenolic compounds than brown rice [25]. Ferulic acid and *p*-coumaric acid are found in the highest quantity in white rice, brown rice and germinated brown rice [25]. Generally, in germinated brown rice (24.78 mg/100 g of flour), insoluble phenolic compounds are 1–2-times more when compared to brown rice (18.47 mg/100 g of flour) [25]. The high levels of phenolic compounds in germinated brown rice are due to the increase in the free forms with alkaline hydrolysis, and this is because of the dismantling of the cell wall during germination [25]. The high levels of insoluble phenolic compounds can enhance the availability of hydrolyzable insoluble phenolic compounds during the germination of brown rice [25].

Amongst other antioxidants that constitute brown rice are the flavonoids. The chemical structure of flavonoids is constituted of a 15-carbon skeleton, which itself is constituted of two aromatic rings interlinked by a heterocyclic ring. The antioxidant activity of the flavonoids stems from the phenolic hydroxyls. Flavones are the most common flavonoids found in brown rice, and tricin is the major flavonoid, accounting for more than 75% of the flavonoids in brown rice [36]. Other flavonoids such as luteolin, apigenin, quercetin, isorhamnetin, kaempferol and myricetin are at relatively low concentrations, as well as isovitexin, naringenin, hesperidin, rutin, luteolin-7-*O*-glucoside, apigenin-7-*O*-glucoside and quercetin-3-*O*-glucoside, amongst others that have been reported [36].

Brown rice also contains sterols present in the bran. The most common sterol is γ-oryzanol, a ferulic acid ester of major phytosterols: campesterol, stigmasterol and β-sitosterol or triterpene alcohols [36]. The γ-oryzanol exhibits several physiological properties including effects on the anthropometry and muscles, cholesterol levels and potential anti-cancer properties [37]. Several analytical methods have been used for the determination of the phytochemical compounds in rice [38,39,40,41,42,43,44,45,46,47] (Table 2).

### Microbial Profiling in Brown Rice

Since the bran and embryo of brown rice are rich in vitamins and fibre, brown rice has the capacity to harbour more microbial association than white rice [48]. During germination, the quality of rice will be improved because the high molecular weight polymers undergo hydrolysis to produce GABA, amino acids, fibres and other bioactive compounds [9]. The germination usually takes place in a warm and humid condition, which favours the growth of microorganisms [49]. The germination of brown rice is initiated when soaking of brown rice occurs. This process involves fermentation because the microbial flora of the environment act upon it after the brown rice is soaked in the water for a certain period of time [50]. Some of these microorganisms can be either harmful or beneficial for consumers [51,52,53,54,55]. For example, some lactic acid bacteria including *Lactobacillus fermentum, Pediococcus pentosaceus* and *Weissella confuse* are detected in germinated brown rice [56]. Table 3 shows the types of microbial association in rice.

## 3. Nutrigenomic Implications of Brown Rice

Similar to other plants [57,58,59,60], although the review of the literature has reported on the health benefits of brown rice, these studies often cannot provide the direct causal relationship between a bioactive compound of brown rice and the observed health benefits. Therefore, it is important to note that these studies should not be over-interpreted because this might be the simplification of the complicated mechanisms in the body that lead to such observed health benefits related to the consumption of brown rice. For example, the review of the literature has shown that brown rice is associated with a wide range of pharmacological properties such as anti-diabetic, anti-cholesterol, anti-hyperlipidemic, cardioprotective and antioxidant [61,62,63,64,65,66,67]. Table 4 shows the summary of some important nutrigenomic mechanisms involved in brown rice

### 3.1. Anti-Diabetic Effect

Type 2 diabetes is a worldwide epidemic affecting millions of people across the world and associated with significant morbidity and mortality. Diet and life style factors play an important role in the pathogenicity of type 2 diabetes. Therapeutic management of the disease is only partially effective, costly and associated with adverse side effects. Therefore, scientists and healthcare professionals are looking for alternative management approaches that are safe, affordable and easily accessible to people, especially those residing in the low and middle income countries. In recent years, a considerable increase in scientific research has been observed regarding the use of brown rice for effective management of diabetes mellitus since it is the main staple food in many parts of the world, especially developing countries of Asia and Africa.

Several population-based studies have shown increased risk of type 2 diabetes associated with the intake of white rice, while higher dietary intake or substitution of white rice with brown rice in the diet may decrease the risk [1,27]. In the same context, results of clinical studies are also encouraging. Recently, a research group in Japan has reported a significant decrease in postprandial glucose level in diabetic patients following consumption of glutinous brown rice for one day [68]. The same group has also reported improved glycaemic control in diabetic patients even after eight weeks of ingestion of glutinous brown rice [69]. Using an open-labelled, randomized cross-over study design, they observed a significant decrease in postprandial plasma glucose, haemoglobin A1c (HbA1c) and glycoalbumin levels in patients who ate glutinous brown rice twice a day compared to those on white rice. Another study of similar duration and dietary intervention on Japanese diabetic patients has also reported decreased levels of postprandial plasma glucose levels and improved endothelial function. However, no significant changes were observed in the HbA1c level [35]. Similarly, a randomized controlled trial on Korean type 2 diabetic patients who followed a brown rice-based vegan diet for 12 weeks have also shown improved glycaemic control (larger reductions in HbA1c level) compared to those who followed the conventional diabetic diet [70].

Several other clinical studies have also reported a decreased glycaemic index and better glycaemic and insulin responses in healthy, diabetic and overweight subjects following consumption of brown rice [71,72,73,74]. All these beneficial effects are mainly attributed to several bioactive compounds present in brown rice. Brown rice has been shown to prevent type 2 diabetes in several studies [68,69,75]. Brown rice has a crucial role in lowering postprandial blood glucose levels in humans [75]. Apart from that, it also helped in weight management and ameliorated glucose and lipid dysmetabolism in individuals with metabolic syndrome. Brown rice contains high amounts of dietary fibre and other polysaccharides such as arabinoxylan and β-glucan. These fibres and polysaccharides help in regulating glucose absorption in the intestine, thus lowering the glycaemic index [76,77]. It also acts as growth substrates for these components to help in the growth of beneficial bacteria in the gut such as *Lactobacillus* and *Bifidobacterium* [78], thus modulating the gut microbial composition and helping in the prevention of diabetes and obesity [75,79]. It is found that in a recent study, there was a significant relationship between the composition of the intestinal microbiota, obesity and type 2 diabetes [75]. Brown rice is proven to have an important effect on the gut microbial composition in humans. This further supports that there is a relationship between the profile and activity of the intestinal microbiota with the anti-obesity and anti-diabetic effects of brown rice [75].

The rice bran in brown rice is rich in γ-oryzanol, which is responsible for many pharmacological properties, such as cholesterol lowering, anti-inflammatory, anti-cancer, anti-diabetic and antioxidant activities. Brown rice ameliorated glucose tolerance and insulin resistance. A lower glycaemic index was observed in healthy (12.1% lower) and diabetic subjects (35.6% lower) due to consumption of brown rice, and this could help to avoid type 2 diabetes and control glycemia, respectively [74]. These effects are due to the rich bioactive content found in brown rice [63]. A study by Sun et al. [1] has proven that consumption of white rice increased the chances of type 2 diabetes, while substituting one third of the daily serving with brown rice lowered the risk of type 2 diabetes in 200,000 subjects. Brown rice that undergoes germination also has differences in its properties, whereby it is less chewy and richer in bioactive compounds [63]. In germinated brown rice, the high content of dietary fibre helps to decrease the glycaemic index by regulating the absorption of glucose in the intestines [76]. Hypoadiponectinemia, which is implicated in reduced insulin sensitivity in diabetes, can be stopped by γ-oryzanol that is found in brown rice [80]. It also acts on pancreatic islets and increases glucose-stimulated insulin secretion [75]. In addition, GABA, another important bioactive present in brown rice, also has shown a similar effect against hypoadiponectinemia [81].

Similarly, acylated steryl glycoside (ASG), a component found in brown rice, regenerates sodium potassium adenosine triphosphatase and homocysteine thiolactonase enzymes with potential to reverse diabetic neuropathy and oxidative changes on biomolecules [82]. It also enhanced the overall metabolic condition in diabetes as a result of the induction of insulin-like growth factor-1 and reduced oxidative stress, which is a problem in type 2 diabetes [83]. The molecular targets of all these bioactive compounds discussed above are not known. However, it is believed that dysregulation of peroxisome proliferator-activated receptors gamma (PPARγ) is linked to the development of metabolic conditions including type 2 diabetes. Thus, PPARs can be a potential target for bioactive compounds, including those present in germinated brown rice. Indeed, a study by Imam et al. has already reported upregulation of PPARγ following treatment of HEP-G2 cells with germinated brown rice bioactive compounds. Upregulation of PPARγ has therapeutic potential in the management of diabetes.

### 3.2. Anti-Dyslipoproteinemia

Dyslipoproteinemia is a group of heterogeneous disorders characterized by elevated plasma cholesterol, triglycerides and lipoproteins level. Dyslipoproteinemia is an important risk factor for an array of clinical conditions including atherosclerosis, cardiovascular diseases and acute pancreatitis [84]. Diet plays an important role in inducing dyslipoproteinemia as evident by the rise in the incidence of the disease due to the intake of modern diets high in fats, sugars and refined grain products. Many studies have demonstrated that brown rice also has anti-dyslipoproteinemia and cholesterol lowering effects in animal models. A study by Shen et al. [85] reported an improved lipid profile (significantly decreased level of triglycerides, total cholesterol, high density lipoprotein and non-high density lipoprotein) in mice fed with pre-germinated brown rice-containing high fat diet for 16 weeks. The authors reported that feeding mice with a high fat diet induced dyslipidemia, which can be successfully averted when the mice were fed a high fat diet supplemented with pre-germinated brown rice [85]. The exact mechanisms are not known; however, this might be achieved by decreasing lipid absorption and synthesis and increasing lipid metabolism. The germinated brown rice extract administration in high fat diet-induced obese mice resulted in a significant reduction in serum triglycerides and total cholesterol levels by suppressing lipogenesis via downregulation of genes involved in lipid synthesis [86].

Another pre-clinical study by Miura et al. [87] reported that feeding hepatoma-bearing rats with white rice resulted in hypercholesteremia, which could be successfully suppressed when the rats were fed with a diet containing germinated brown rice. They probably do so by upregulating cholesterol catabolism. Other studies have also reported anti-dyslipoproteinemia and cholesterol lowering effects of germinated brown rice [88,89]. Human clinical studies evaluating the effects of germinated brown rice on dyslipoproteinemia are limited. In a clinical study involving sixty Vietnamese women (aged 45–65 years) with impaired glucose tolerance, the impact of germinated brown rice and white rice intake on blood glucose and lipid profile was evaluated. Following four months of intervention, Bui et al. [90] observed an improvement in blood glucose and lipid level in the pre-germinated brown rice diet group compared to the white rice group.

Similarly, a randomized control trial on 11 diabetic patients also reported a significant reduction in serum total cholesterol and triglyceride level following consumption of pre-germinated brown rice for 14 weeks compared to white rice group [91]. However, no such improvement in serum lipid profile and other metabolic parameters was observed in healthy volunteers who followed either a white rice diet or a white rice plus germinated brown rice diet (1:1, *w*/*w*) for 11–13 months [92]. However, this may be due to the presence of white rice in the diet, which diminishes the beneficial effects of germinated brown rice [72]. Hypercholesterolemia induced by hepatoma growth can be suppressed by means of upregulating cholesterol metabolism. Germinated brown rice also has a greater effect on the restorative effects on cholesterol levels compared to brown rice. This proves that germinated brown rice has a greater impact on high blood cholesterol [88]. All these beneficial activities of brown rice are mainly attributed to the presence of the high concentration of various biologically-active components such as GABA, dietary fibre, γ-oryzanol and other antioxidants in brown rice that help in preventing hyperlipidaemia. The risk of atherogenesis and coronary artery disease through its protection against LDL oxidation can be reduced by the antioxidant contents in brown rice and germinated brown rice [10,93].

### 3.3. Anti-Cancer Effect

Recent studies have reported the chemo-preventive and anticancer potential of some biologically-active molecules present in germinated brown rice. These molecules can prevent or suppress cancer development. Chemopreventive activities of germinated rough rice have been demonstrated in a recent study [94]. Using azoxymethane, colon cancer was induced in six-week-old male Sprague-Dawley rats followed by oral administration of either control diet or different doses of germinated rough rice crude extract (2000, 1000 and 5000 mg/kg body weight) once daily for eight weeks. The study showed a dose-dependent reduction in the size and number of aberrant crypt foci formation and β-catenin expression in rats fed with germinated rough rice crude extract.

Similarly, a study by Latifah et al. [95] also showed a significant decrease in aberrant crypt foci formation and β-catenin and cox-2 expression when azoxymethane-induced colon cancer rats were fed with different doses of germinated brown rice (2.5, 5 and 10 g/kg body weight). From germinated brown rice, GABA-enhanced parts were extracted, and they portrayed inhibitory action on the reproduction of some cancer cells and a stimulatory action on immune responses. GABA-enriched extracts from germinated brown rice had also been shown to inhibit effects on leukemic cells’ proliferation and to stimulate cancer cells in terms of apoptosis [96]. GABA may also play a role in protecting smokers from pulmonary adenocarcinoma due to the reported tumour suppression activity in small airway epithelia [97]. Besides GABA, other bioactive compounds such as tocopherols and tocotrienols present in brown rice may also exhibit anticancer potential [98]. All these studies suggest the potential role of germinated brown rice in cancer prevention. However, further epidemiological and clinical studies are required in order to utilize germinated brown rice as a staple food in cancer prevention activity and for its inhibitory effect. The leukaemia cells that were treated with germinated brown rice extract showed greater DNA fragmentation compared to leukaemia cells treated with brown rice [11]. Apart from that, immunoregulatory activities found in germinated brown rice enhance the cell proliferation of mesenteric lymph node cells in vitro and also increase murine splenic B, T-helper cell subpopulations and nitric oxide c- interferon production [96].

### 3.4. Lowering Cholesterol

GABA found in brown rice also helps to nourish blood vessels, regulate insulin secretion, avoid increasing blood cholesterol, reduce emotional unrest, improvement from stroke, better the kidney and liver function and prevent chronic alcohol disease [11]. The rice bran oil (RBO) found in brown rice can help to reduce the atherogenic level and increase HDL cholesterol. The cholesterol-reducing activity induced by RBO was due to the decreased absorption-reabsorption of cholesterol and the interference of the plant sterols in cholesterol metabolism. When the unsaponifiable matters of rice bran were fed to hamsters in a study, the faecal fat and neutral sterol excretion was greater. This shows that there is a decrease in fat digestibility [11].

### 3.5. Cardio-Protective Effect

Cardiovascular disease (CVD) includes diseases of the heart and circulatory system including angina, hypertension, heart attack, congenital heart disease and stroke. On a global scale, CVD is the most common cause of death with an estimated 17.7 million people dying because of CVD in 2015 [99]. To reduce the risk and increase protection against CVD, effective nutritional intervention has always been a focus of public health strategies. In this regard, brown rice and its bioactive compounds have been reported to possess anti-hyperlipidemic, anti-hypercholesteraemic and antihypertensive potential and thus have a role in preventing CVD.

Recently, a randomized cross-over clinical trial was conducted to evaluate the effect of brown rice consumption on inflammatory markers and cardiovascular risk factors [100]. Forty non-menopausal overweight or obese women (BMI > 25) were recruited and divided into two groups. Participants in both groups were asked to consume 150 g cooked brown rice or white rice for six weeks, followed by a two-week wash out period and switching over to an alternate diet for six weeks. Results of the study revealed that consumption of brown rice can significantly reduce inflammatory markers (CRP) and other risk factors (weight, BMI, waist, diastolic blood pressure) associated with CVD.

Another clinical study on healthy female university students revealed that ingestion of brown rice as a staple food for 10 weeks improved general health and prevented hyperlipidaemia, thus protecting against CVD [101]. Several pre-clinical studies have also reported anti-hypertensive effects of germinated brown rice in spontaneously hypertensive rats [102,103]. Mechanistic studies revealed that the antihypertensive effects may be due to the presence of several bioactive compounds in brown rice such as GABA, dietary fibres and ferulic acids [104,105]. On this basis, germinated brown rice can be a good choice as a staple diet or functional food to prevent CVD.

### 3.6. Antioxidant Effect

Brown rice contains many types of phenolic acids, which are well known for their antioxidant activities and one of the most common antioxidants in our diet. They can protect cells against oxidative damage, thereby reducing the risk of diseases associated with oxidative damage. Prevention of diseases such as cardiovascular disease, type 2 diabetes, obesity and cancer is possible due to the high antioxidant levels found in brown rice. The phenolic acids from brown rice also are assumed to contain chemopreventive properties for breast and colon cancer [106]. Based on the sensory attributes, the whole rice grain is harder to chew and has less taste qualities. Thus, pre-germinated rice is favoured. Brown rice is first soaked in water to initiate germination and contains a higher nutritional value. It is also shown that pre-germinated brown rice increases mental health and immunity. It also helps to prevent diabetic decline [107]. Hepatic fibrosis is one of the most prevalent health problems, and it can be prevented by consuming brown rice. Ferulic acid, *p*-coumaric acid, γ-oryzanol, γ-tocotrienol, GABA and other components in pre-germinated brown rice can decrease liver inflammation and fibrosis and hence reduce the risk of liver cirrhosis and cancer [108].

## 4. Conclusions and Future Research

Our review has highlighted that brown rice contains certain bioactive phytochemical compounds that might be associated with some important nutrigenomic implications. Therefore, brown rice has received increasing attention from consumers who are health-conscious. In addition, our review also suggests that there are several opportunities for the food industry to develop a wide range of food products using brown rice as the main ingredient. Similar to other plants [59,60,109], future research should be designed to screen for the individual bioactive components that might be associated with the nutrigenomic implications of brown rice.

## Figures and Tables

**Table 1:** Summary of the major phytochemical composition of brown rice.

Family	Compounds
Phenolics	Gallic acid, protocatechuic acid, *p*-hydroxybenzoic acid, vanillic acid, syringic acid, chlorogenic acid, caffeic acid, *p*-coumaric acid, sinapic acid, ferulic acid, cinnamic acid, ellagic acid
Flavonoids	Luteolin, apigenin, tricin, quercetin, kaempferol, isorhamnetin, myricetin
Anthocyanins and proanthocyanins	Peonidin-3-*O*-glucoside, cyanidin-3-*O*-glucoside, cyanidin-3-*O*-galactoside, cyanidin-3-*O*-rutinoside, catechin, epicatechin
Vitamins	Tocopherols, tocotrienols, B vitamins (B1, B3, B6)
Amino acids	Alanine, arginine, aspartic acid, cystine, glutamic acid, glycine, histidine, isoleucine, leucine, lysine, methionine, phenylalanine, proline, serine, threonine, tryptophan, tyrosine, valine
Phytosterols	Stigmasterol, stigmastanol, β-sitosterol, campesterol, δ5-avenasterol, δ7-avenasterol
γ-Oryzanol	Cycloartanyl ferulate, 24-methylene cycloartanyl ferulate, campesteryl ferulate, β-sitosteryl ferulate
Others	Dietary fibre, phytic acid, minerals

**Table 2:** Summary of analytical methods used to identify the phytochemical compounds in rice.

Phytochemical Compounds in Rice	Analytical Methods	References
Phenolic acids	Microwave-assisted extraction (MAE) Ultrasound-assisted extraction (UAE)	Sato et al. (2004) [38]
Antioxidants	Microwave-assisted extraction (MAE) Ultrasound-assisted extraction (UAE)	Sato et al. (2004) [38]
Anthocyanins and proanthocyanins	UV-visible spectroscopy	Sato et al. (2004) [38]
Dietary fibre	Enzymatic-gravimetric method	Tiansawang et al. (2016) [39]
Functional lipid	Gravimetric method	Zhou et al. (2003) [40]
Essential amino acid	HPLC method	Naomi et al. (2014) [41]
Phytosterols	Gas chromatography	Zubair et al. (2012). [42]
Flavonoids	Fluorescent DCF	Srisawat et al. (2011) [43]
Tocopherols and tocotrienols	Fluorescent DCF	Srisawat et al. (2011) [43]
Minerals	Ashing method	Horwitz (2000) [44]
Gamma aminobutyric acid (GABA)	Amino acid auto analyser	Cao et al. (2015) [45]
γ-oryzanol	Reversed-phase HPLC method	Xu and Godber (1999) [47]
Phytic acid	UV-Vis spectroscopy	Perera et al. (2018) [46]

**Table 3:** Summary of major microbial association in rice.

Group	Microbes	Microbial Association	References
Gram positive bacteria	*Brevibacillus laterosporus*, *Brevibacillus brevis*, *Brevibacterium* spp.	Production of amino acids	Cottyn et al. [51]
	*Cellulomonas flavigena*	Degradation of cellulose	Cottyn et al. [51]
	*Bacillus thuringiensis* and *Bacillus cereus*	Production of enterotoxin	Kim et al. [52]
	*Staphylococcus saprophyticus*	Food-borne pathogen	Cottyn et al. [51]
Fungi	*Monascus purpureus*	Production of red pigment	Pengnoi et al. [53]
	*Fusarium fujikuroi*, *Aspergillus flavus* and *Candida*	Production of toxin	Tanaka et al. [54]
Yeast	*Torulopsis etchellsii*, *Hansenula anomala*, *Trichosporon pullulans*, *Geotrichum candidum*, *Saccharomyces* sp.	An increase in the essential amino acids; a decrease in phytic acid and enzyme inhibitors	Panneerselvam et al. [2]; Shortt [55]

**Table 4:** Summary of some important nutrigenomic mechanisms involved in brown rice.

Property	Potential Underlying Nutrigenomic Mechanism	References
Antioxidative	An increase in antioxidant status and a reduction in oxidative stress via v-akt murine thyromoma viral oncogene (AKT), nuclear factor beta (NF-Kβ), mitogen activated protein kinase (p38 MAPK), c-Jun N-terminal kinase (JNK), extracellular signal-regulated kinase (ERK1/2), p53 tumour suppressor genes, catalase, insulin-like growth factor 2 (IGF2) and superoxide dismutase (SOD)	Azmi et al. (2013) [61]; Imam et al. (2013) [62]; Imam et al. (2012a) [63]; Imam et al. (2012b) [64]
Anti-hyperglycemia	A decrease in the level of blood glucose via the suppression of fbp and pck genes, which are gluconeogenic	Imam and Ismail [62]
Anti-hypocholesterolaemia	A decrease in low density lipoprotein (LDL) and total cholesterol, as well as an increase in high density lipoprotein (HDL) via the transcriptional regulation of hepatic LDL receptor, lipoprotein lipase (LPL), adiponectin, peroxisome proliferator-activator receptor (PPAR) γ, ATP binding cassette (ABCA) 1, AKT and apolipoprotein genes	Imam et al. (2013) [66]; Imam et al. [67]

## References

[1] Sun Q., Spiegelman D., van Dam R.M., Holmes M.D., Malik V.S., Willett W.C., Hu F.B. (2010). White rice, brown rice, and risk of type 2 diabetes in US men and women. Arch. Intern. Med..

[2] Panneerselvam P., Binodh A.K., Kumar U., Sugitha T., Anandan A., Manickavasagan A., Santhakumar C., Venkatachalapathy N. (2017). Microbial association in brown rice and their influence on human health. Brown Rice.

[3] Fairhurst T., Dobermann A. (2002). Rice in the global food supply. Better Crops Int..

[4] Zareiforoush H., Minaei S., Alizadeh M.R., Banakar A. (2016). Qualitative classification of milled rice grains using computer vision and metaheuristic techniques. J. Food Sci. Technol..

[5] Kale S.J., Jha S.K., Jha G.K., Sinha J.P., Lal S.B. (2015). Soaking induced changes in chemical composition, glycemic index and starch characteristics of basmati rice. Rice Sci..

[6] Chen H., Siebenmorgen T.J., Griffin K. (1998). Quality characteristics of long-grain rice milled in two commercial systems. Cereal Chem. J..

[7] Vetha Varshini P., Azhagu Sundharam K., Vijay Praveen P. (2013). Brown rice—Hidden nutrients. J. Biosci. Tech..

[8] Liu L., Guo J., Zhang R., Wei Z., Deng Y., Guo J., Zhang M. (2015). Effect of degree of milling on phenolic profiles and cellular antioxidant activity of whole brown rice. Food Chem..

[9] Ohtsubo K.I., Suzuki K., Yasui Y., Kasumi T. (2005). Bio-functional components in the processed pre-germinated brown rice by a twin-screw extruder. J. Food Compos. Anal..

[10] Wu F., Yang N., Touré A., Jin Z., Xu X. (2013). Germinated brown rice and its role in human health. Crit. Rev. Food Sci. Nutr..

[11] Cho D.H., Lim S.T. (2016). Germinated brown rice and its bio-functional compounds. Food Chem..

[12] Foster-Powell K., Holt S.H., Brand-Miller J.C. (2002). International table of glycemic index and glycemic load values: 2002. Am. J. Clin. Nutr..

[13] Umadevi M., Pushpa R., Sampathkumar K., Bhowmik D. (2012). Rice—Traditional medicinal plant in India. J. Pharmacogn. Phytochem..

[14] Burlando B., Cornara L. (2014). Therapeutic properties of rice constituents and derivatives (*Oryza sativa* L.): A review update. Trends Food Sci. Technol..

[15] Hegde S., Yenagi N., Kasturiba B. (2013). Indigenous knowledge of the traditional and qualified ayurveda practitioners on the nutritional significance and use of red rice in medications. Indian J. Tradit. Knowl..

[16] Isaac R.S.R., Nair A.S., Varghese E., Chavali M. (2012). Phytochemical, antioxidant and nutrient analysis of medicinal rice (*Oryza sativa* L.) varieties found in south India. Adv. Sci. Lett..

[17] Okarter N., Liu R.H. (2010). Health benefits of whole grain phytochemicals. Crit. Rev. Food Sci. Nutr..

[18] Babu P.D., Subhasree R., Bhakyaraj R., Vidhyalakshmi R. (2009). Brown rice-beyond the color reviving a lost health food—A review. Am.-Eurasian J. Agron..

[19] Gong E.S., Luo S.J., Li T., Liu C.M., Zhang G.W., Chen J., Zeng Z.C., Liu R.H. (2017). Phytochemical profiles and antioxidant activity of brown rice varieties. Food Chem..

[20] Tan B.L., Norhaizan M.E. (2017). Scientific evidence of rice by-products for cancer prevention: Chemopreventive properties of waste products from rice milling on carcinogenesis in vitro and in vivo. BioMed Res. Int..

[21] Liu R.H. (2007). Whole grain phytochemicals and health. J. Cereal Sci..

[22] Adom K.K., Liu R.H. (2002). Antioxidant activity of grains. J. Agric. Food Chem..

[23] Adom K.K., Sorrells M.E., Liu R.H. (2005). Phytochemicals and antioxidant activity of milled fractions of different wheat varieties. J. Agric. Food Chem..

[24] Martínez I., Lattimer J.M., Hubach K.L., Case J.A., Yang J., Weber C.G., Louk J.A., Rose D.J., Kyureghian G., Peterson D.A. (2013). Gut microbiome composition is linked to whole grain-induced immunological improvements. ISME J..

[25] Tian S., Nakamura K., Kayahara H. (2004). Analysis of phenolic compounds in white rice, brown rice, and germinated brown rice. J. Agric. Food Chem..

[26] Cáceres P.J., Peñas E., Martinez-Villaluenga C., Amigo L., Frias J. (2017). Enhancement of biologically active compounds in germinated brown rice and the effect of sun-drying. J. Cereal Sci..

[27] Adebamowo S.N., Eseyin O., Yilme S., Adeyemi D., Willett W.C., Hu F.B., Spiegelman D., Adebamowo C.A. (2017). A mixed-methods study on acceptability, tolerability, and substitution of brown rice for white rice to lower blood glucose levels among Nigerian adults. Front. Nutr..

[28] Patil S.B., Khan M.K. (2011). Germinated brown rice as a value added rice product: A review. J. Food Sci. Technol..

[29] Vogt T.M., Ziegler R.G., Graubard B.I., Swanson C.A., Greenberg R.S., Schoenberg J.B., Swanson G.M., Hayes R.B., Mayne S.T. (2003). Serum selenium and risk of prostate cancer in U.S. blacks and whites. Int. J. Cancer.

[30] Anderson J.W., Hanna T.J. (1999). Whole grains and protection against coronary heart disease: What are the active components and mechanisms?. Am. J. Clin. Nutr..

[31] Jiamyangyuen S., Ooraikul B. (2008). The physico-chemical, eating and sensorial properties of germinated brown rice. J. Food Agric. Environ..

[32] Aune D., Chan D.S.M., Lau R., Vieira R., Greenwood D.C., Kampman E., Norat T. (2011). Dietary fibre, whole grains, and risk of colorectal cancer: Systematic review and dose-response meta-analysis of prospective studies. Br. Med. J..

[33] Aune D., Chan D.S., Greenwood D.C., Vieira A.R., Rosenblatt D.A., Vieira R., Norat T. (2012). Dietary fiber and breast cancer risk: A systematic review and meta-analysis of prospective studies. Ann. Oncol..

[34] Verschoyle R.D., Greaves P., Cai H., Edwards R.E., Steward W.P., Gescher A.J. (2007). Evaluation of the cancer chemopreventive efficacy of rice bran in genetic mouse models of breast, prostate and intestinal carcinogenesis. Br. J. Cancer.

[35] Kondo K., Morino K., Nishio Y., Ishikado A., Arima H., Nakao K., Nakagawa F., Nikami F., Sekine O., Nemoto K.I. (2017). Fiber-rich diet with brown rice improves endothelial function in type 2 diabetes mellitus: A randomized controlled trial. PLoS ONE.

[36] Goufo P., Trindade H. (2014). Rice antioxidants: Phenolic acids, flavonoids, anthocyanins, proanthocyanidins, tocopherols, tocotrienols, gamma-oryzanol, and phytic acid. Food Sci. Nutr..

[37] Cicero A.F., Gaddi A. (2001). Rice bran oil and gamma-oryzanol in the treatment of hyperlipoproteinaemias and other conditions. Phytother. Res..

[38] Sato S., Soga T., Nishioka T., Tomita M. (2004). Simultaneous determination of the main metabolites in rice leaves using capillary electrophoresis mass spectrometry and capillary electrophoresis diode array detection. Plant J..

[39] Tiansawang K., Luangpituksa P., Varanyanond W., Hansawasdi C. (2016). GABA production, antioxidant activity in some germinated dietary seeds and the effect of cooking on their GABA content. Food Sci. Technol..

[40] Zhou Z., Blanchard C., Helliwell S., Robards K. (2003). Fatty acid composition of three rice varieties following storage. J. Cereal Sci..

[41] Eshun Naomi A., Emmanuel A., Barimah J., VM D., Van Twisk C. (2015). Amino acid profiles of some varieties of rice, soybean and groundnut grown. J. Food Process. Technol..

[42] Zubair M., Anwar F., Ashraf M., Uddin M.K. (2012). Characterization of high-value bioactives in some selected varieties of Pakistani Rice (*Oryza sativa* L.). Int. J. Mol. Sci..

[43] Srisawat U., Panunto W., Kaendee N., Tanuchit S., Itharat A., Lerdvuthisopon N., Hansakul P. (2010). Determination of phenolic compounds, flavonoids, and antioxidant activities in water extracts of Thai red and white rice cultivars. J. Med. Assoc. Thail..

[44] Horwitz W. (2000). Official Methods of Analysis of AOAC International.

[45] Cao Y., Jia F., Han Y., Liu Y., Zhang Q. (2015). Study on the optimal moisture adding rate of brown rice during germination by using segmented moisture conditioning method. J. Food Sci. Technol..

[46] Perera I., Seneweera S., Hirotsu N. (2018). Manipulating the phytic acid content of rice grain toward improving micronutrient bioavailability. Rice.

[47] Xu Z., Godber J.S. (1999). Purification and identification of components of γ-oryzanol in rice bran oil. J. Agric. Food Chem..

[48] Cui L., Pan Z., Yue T., Atungulu G.G., Berrios J. (2010). Effect of ultrasonic treatment of brown rice at different temperatures on cooking properties and quality. Cereal Chem. J..

[49] Lu Z.H., Zhang Y., Li L.T., Curtis R.B., Kong X.L., Fulcher R.G., Zhang G., Cao W. (2010). Inhibition of microbial growth and enrichment of gamma-aminobutyric acid during germination of brown rice by electrolyzed oxidizing water. J. Food Prot..

[50] Puri S., Dhillon B., Sodhi N.S. (2014). Effect of degree of milling (Dom) on overall quality of rice—A review. Int. J. Adv. Biotechnol. Res..

[51] Cottyn B., Regalado E., Lanoot B., De Cleene M., Mew T.W., Swings J. (2001). Bacterial populations associated with rice seed in the tropical environment. Phytopathology.

[52] Kim B., Bang J., Kim H., Kim Y., Kim B.S., Beuchat L.R., Ryu J.H. (2014). *Bacillus cereus* and *Bacillus thuringiensis* spores in Korean rice: Prevalence and toxin production as affected by production area and degree of milling. Food Microbiol..

[53] Pengnoi P., Mahawan R., Khanongnuch C., Lumyong S. (2017). Antioxidant properties and production of monacolin k, citrinin, and red pigments during solid state fermentation of purple rice (*Oryzae sativa*) varieties by *Monascus purpureus*. Czech J. Food Sci..

[54] Tanaka K., Sago Y., Zheng Y., Nakagawa H., Kushiro M. (2007). Mycotoxins in rice. Int. J. Food Microbiol..

[55] Shortt C. (1998). Living it up for dinner. Chem. Ind..

[56] Kim K.-S., Kim B.-H., Kim M.-J., Han J.-K., Kum J.-S., Lee H.-Y. (2012). Quantitative microbiological profiles of brown rice and germinated brown rice. Food Sci. Biotechnol..

[57] Ma Z.F., Zhang H. (2017). Phytochemical constituents, health benefits, and industrial applications of grape seeds: A mini-review. Antioxidants.

[58] Ma Z.F., Lee Y.Y. (2016). Virgin coconut oil and its cardiovascular health benefits. Nat. Prod. Commun..

[59] Zhang H., Ma Z.F. (2018). Phytochemical and pharmacological poperties of *Capparis spinosa* as a medicinal plant. Nutrients.

[60] Cao Y., Ma Z., Zhang H., Jin Y., Zhang Y., Hayford F. (2018). Phytochemical properties and nutrigenomic implications of yacon as a potential source of prebiotic: Current evidence and future directions. Foods.

[61] Azmi N.H., Ismail N., Imam M.U., Ismail M. (2013). Ethyl acetate extract of germinated brown rice attenuates hydrogen peroxide-induced oxidative stress in human SH-SY5Y neuroblastoma cells: Role of anti-apoptotic, pro-survival and antioxidant genes. BMC Complement. Altern. Med..

[62] Imam M.U., Ismail M. (2013). Nutrigenomic effects of germinated brown rice and its bioactives on hepatic gluconeogenic genes in type 2 diabetic rats and HEPG2 cells. Mol. Nutr. Food Res..

[63] Imam M.U., Musa S.N.A., Azmi N.H., Ismail M. (2012). Effects of white rice, brown rice and germinated brown rice on antioxidant status of type 2 diabetic rats. Int. J. Mol. Sci..

[64] Imam M.U., Ismail M., Omar A.R. (2012). Nutrigenomic effects of germinated brown rice bioactives on antioxidant genes. Free Radic. Biol. Med..

[65] Imam M.U., Azmi N.H., Ismail M. (2012). Upregulation of superoxide dismutase gene is involved in antioxidant effects of germinated rown rice. Free Radic. Biol. Med..

[66] Imam M.U., Ismail M., Omar A.R., Ithnin H. (2013). The hypocholesterolemic effect of germinated brown rice involves the upregulation of the apolipoprotein A1 and low-density lipoprotein receptor genes. J. Diabetes Res..

[67] Imam M.U., Ishaka A., Ooi D.-J., Zamri N.D.M., Sarega N., Ismail M., Esa N.M. (2014). Germinated brown rice regulates hepatic cholesterol metabolism and cardiovascular disease risk in hypercholesterolaemic rats. J. Funct. Foods.

[68] Terashima Y., Nagai Y., Kato H., Ohta A., Tanaka Y. (2017). Eating glutinous brown rice for one day improves glycemic control in Japanese patients with type 2 diabetes assessed by continuous glucose monitoring. Asia Pac. J. Clin. Nutr..

[69] Nakayama T., Nagai Y., Uehara Y., Nakamura Y., Ishii S., Kato H., Tanaka Y. (2017). Eating glutinous brown rice twice a day for 8 weeks improves glycemic control in Japanese patients with diabetes mellitus. Nutr. Diabetes.

[70] Lee Y.M., Kim S.A., Lee I.K., Kim J.G., Park K.G., Jeong J.Y., Jeon J.H., Shin J.Y., Lee D.H. (2016). Effect of a brown rice based vegan diet and conventional diabetic diet on glycemic control of patients with type 2 diabetes: A 12-week randomized clinical trial. PLoS ONE.

[71] Shobana S., Lakshmipriya N., Bai M.R., Gayathri R., Ruchi V., Sudha V., Malleshi N.G., Krishnaswamy K., Henry C.K., Anjana R.M. (2017). Even minimal polishing of an Indian parboiled brown rice variety leads to increased glycemic responses. Asia Pac. J. Clin. Nutr..

[72] Ito Y., Mizukuchi A., Kise M., Aoto H., Yamamoto S., Yoshihara R., Yokoyama J. (2005). Postprandial blood glucose and insulin responses to pre-germinated brown rice in healthy subjects. J. Med. Investig..

[73] Mohan V., Spiegelman D., Sudha V., Gayathri R., Hong B., Praseena K., Anjana R.M., Wedick N.M., Arumugam K., Malik V. (2014). Effect of brown rice, white rice, and brown rice with legumes on blood glucose and insulin responses in overweight Asian Indians: A randomized controlled trial. Diabetes Technol. Ther..

[74] Panlasigui L.N., Thompson L.U. (2006). Blood glucose lowering effects of brown rice in normal and diabetic subjects. Int. J. Food Sci. Nutr..

[75] Kozuka C., Yabiku K., Takayama C., Matsushita M., Shimabukuro M. (2013). Natural food science based novel approach toward prevention and treatment of obesity and type 2 diabetes: Recent studies on brown rice and gamma-oryzanol. Obes. Res. Clin. Pract..

[76] Ou S., Kwok K., Li Y., Fu L. (2001). In vitro study of possible role of dietary fiber in lowering postprandial serum glucose. J. Agric. Food Chem..

[77] Seki T., Nagase R., Torimitsu M., Yanagi M., Ito Y., Kise M., Mizukuchi A., Fujimura N., Hayamizu K., Ariga T. (2005). Insoluble fiber is a major constituent responsible for lowering the post-prandial blood glucose concentration in the pre-germinated brown rice. Biol. Pharm. Bull..

[78] Jaskari J., Kontula P., Siitonen A., Jousimies-Somer H., Mattila-Sandholm T., Poutanen K. (1998). Oat beta-glucan and xylan hydrolysates as selective substrates for *Bifidobacterium* and *Lactobacillus* strains. Appl. Microbiol. Biotechnol..

[79] Benno Y., Endo K., Miyoshi H., Okuda T., Koishi H., Mitsuoka T. (1989). Effect of rice fiber on human fecal microflora. Microbiol. Immunol..

[80] Nagasaka R., Yamsaki T., Uchida A., Ohara K., Ushio H. (2011). Gamma-oryzanol recovers mouse hypoadiponectinemia induced by animal fat ingestion. Phytomedicine.

[81] Ohara K., Kiyotani Y., Uchida A., Nagasaka R., Maehara H., Kanemoto S., Hori M., Ushio H. (2011). Oral administration of gamma-aminobutyric acid and gamma-oryzanol prevents stress-induced hypoadiponectinemia. Phytomedicine.

[82] Usuki S., Ito Y., Morikawa K., Kise M., Ariga T., Rivner M., Yu R.K. (2007). Effect of pre-germinated brown rice intake on diabetic neuropathy in streptozotocin-induced diabetic rats. Nutr. Metab..

[83] Usuki S., Tsai Y.Y., Morikawa K., Nonaka S., Okuhara Y., Kise M., Yu R.K. (2011). IGF-1 induction by acylated steryl beta-glucosides found in a pre-germinated brown rice diet reduces oxidative stress in streptozotocin-induced diabetes. PLoS ONE.

[84] Nordestgaard B.G., Varbo A. (2014). Triglycerides and cardiovascular disease. Lancet.

[85] Shen K.-P., Hao C.-L., Yen H.-W., Chen C.-Y., Chen J.-H., Chen F.-C., Lin H.-L. (2016). Pre-germinated brown rice prevented high fat diet induced hyperlipidemia through ameliorating lipid synthesis and metabolism in C57BL/6J mice. J. Clin. Biochem. Nutr..

[86] Ho J.N., Son M.E., Lim W.C., Lim S.T., Cho H.Y. (2012). Anti-obesity effects of germinated brown rice extract through down-regulation of lipogenic genes in high fat diet-induced obese mice. Biosci. Biotechnol. Biochem..

[87] Miura D., Ito Y., Mizukuchi A., Kise M., Aoto H., Yagasaki K. (2006). Hypocholesterolemic action of pre-germinated brown rice in hepatoma-bearing rats. Life Sci..

[88] Roohinejad S., Omidizadeh A., Mirhosseini H., Saari N., Mustafa S., Yusof R.M., Hussin A.S., Hamid A., Abd Manap M.Y. (2010). Effect of pre-germination time of brown rice on serum cholesterol levels of hypercholesterolaemic rats. J. Sci. Food Agric..

[89] Albarracin M., Weisstaub A.R., Zuleta A., Drago S.R. (2016). Extruded whole grain diets based on brown, soaked and germinated rice. Effects on cecum health, calcium absorption and bone parameters of growing Wistar rats. Part I. Food Funct..

[90] Bui T.N., Le T.H., Nguyen D.H., Tran Q.B., Nguyen T.L., Le D.T., Do V.A., Vu A.L., Aoto H., Okuhara Y. (2014). Pre-germinated brown rice reduced both blood glucose concentration and body weight in Vietnamese women with impaired glucose tolerance. J. Nutr. Sci. Vitaminol..

[91] Hsu T.F., Kise M., Wang M.F., Ito Y., Yang M.D., Aoto H., Yoshihara R., Yokoyama J., Kunii D., Yamamoto S. (2008). Effects of pre-germinated brown rice on blood glucose and lipid levels in free-living patients with impaired fasting glucose or type 2 diabetes. J. Nutr. Sci. Vitaminol..

[92] Morita H., Uno Y., Umemoto T., Sugiyama C., Matsumoto M., Wada Y., Ishizuka T. (2004). Effect of gamma-aminobutyric acid-rich germinated brown rice on indexes of life-style related diseases. Nihon Ronen Igakkai Zasshi.

[93] Wu C., Ye Z., Li H., Wu S., Deng D., Zhu Y., Wong M. (2012). Do radial oxygen loss and external aeration affect iron plaque formation and arsenic accumulation and speciation in rice?. J. Exp. Bot..

[94] Saki E., Saiful Yazan L., Mohd Ali R., Ahmad Z. (2017). Chemopreventive effects of germinated rough rice crude extract in inhibiting azoxymethane-induced aberrant crypt foci formation in sprague-dawley rats. BioMed Res. Int..

[95] Latifah S.Y., Armania N., Tze T.H., Azhar Y., Nordiana A.H., Norazalina S., Hairuszah I., Saidi M., Maznah I. (2010). Germinated brown rice (GBR) reduces the incidence of aberrant crypt foci with the involvement of beta-catenin and COX-2 in azoxymethane-induced colon cancer in rats. Nutr. J..

[96] Oh C.H., Oh S.H. (2004). Effects of germinated brown rice extracts with enhanced levels of GABA on cancer cell proliferation and apoptosis. J. Med. Food.

[97] Schuller H.M., Al-Wadei H.A., Majidi M. (2008). Gamma-aminobutyric acid, a potential tumor suppressor for small airway-derived lung adenocarcinoma. Carcinogenesis.

[98] Har C.H., Keong C.K. (2005). Effects of tocotrienols on cell viability and apoptosis in normal murine liver cells (BNL CL.2) and liver cancer cells (BNL 1ME A.7R.1), in vitro. Asia Pac. J. Clin. Nutr..

[99] Go A.S., Mozaffarian D., Roger V.L., Benjamin E.J., Berry J.D., Blaha M.J., Dai S., Ford E.S., Fox C.S., Franco S. (2014). Heart Disease and Stroke Statistics—2014 Update: A Report From the American Heart Association. Circulation.

[100] Kazemzadeh M., Safavi S.M., Nematollahi S., Nourieh Z. (2014). Effect of brown rice consumption on inflammatory marker and cardiovascular risk factors among overweight and obese non-menopausal female adults. Int. J. Prev. Med..

[101] Ebizuka H., Sasaki C., Kise M., Arita M. (2007). Effects of retort pouched rice containing pre-germinated brown rice on daily nutrition and physical status in healthy subjects. J. Integr. Study Diet. Habits.

[102] Ebizuka H., Ihara M., Arita M. (2009). Antihypertensive effect of pre-germinated brown rice in spontaneously hypertensive rats. Food Sci. Technol. Res..

[103] Choi H.-D., Kim Y.-S., Choi I.-W., Park Y.-K., Park Y.-D. (2006). Hypotensive effect of germinated brown rice on spontaneously hypertensive rats. Korean J. Food Sci. Technol..

[104] Suzuki A., Kagawa D., Fujii A., Ochiai R., Tokimitsu I., Saito I. (2002). Short- and long-term effects of ferulic acid on blood pressure in spontaneously hypertensive rats. Am. J. Hypertens..

[105] Ohsaki Y., Shirakawa H., Koseki T., Komai M. (2008). Novel effects of a single administration of ferulic acid on the regulation of blood pressure and the hepatic lipid metabolic profile in stroke-prone spontaneously hypertensive rats. J. Agric. Food Chem..

[106] Shao Y., Bao J. (2015). Polyphenols in whole rice grain: Genetic diversity and health benefits. Food Chem..

[107] Sakamoto S., Hayashi T., Hayashi K., Murai F., Hori M., Kimoto K., Murakami K. (2007). Pre-germinated brown rice could enhance maternal mental health and immunity during lactation. Eur. J. Nutr..

[108] Wunjuntuk K., Kettawan A., Rungruang T., Charoenkiatkul S. (2016). Anti-fibrotic and anti-inflammatory effects of parboiled germinated brown rice (*Oryza sativa* ‘KDML 105′) in rats with induced liver fibrosis. J. Funct. Foods.

[109] Zhang H., Ma Z.F., Luo X., Li X. (2018). Effect of mulberry fruit (*Morus alba* L.) consumption on health outcomes: A mini-review. Antioxidants.

